# Amino acid-dependent control of mTORC1 signaling: a variety of regulatory modes

**DOI:** 10.1186/s12929-020-00679-2

**Published:** 2020-08-17

**Authors:** Terunao Takahara, Yuna Amemiya, Risa Sugiyama, Masatoshi Maki, Hideki Shibata

**Affiliations:** Department of Applied Biosciences, Graduate School of Bioagricultural Sciences, Nagoya University, Furo-cho, Chikusa-ku, Nagoya, Aichi 464-8601 Japan

**Keywords:** mTOR, mTORC1, Amino acids, Rag GTPases, Rheb GTPase, Intracellular Ca^2+^ concentration

## Abstract

The mechanistic target of rapamycin complex 1 (mTORC1) is an essential regulator of cell growth and metabolism through the modulation of protein and lipid synthesis, lysosome biogenesis, and autophagy. The activity of mTORC1 is dynamically regulated by several environmental cues, including amino acid availability, growth factors, energy levels, and stresses, to coordinate cellular status with environmental conditions. Dysregulation of mTORC1 activity is closely associated with various diseases, including diabetes, cancer, and neurodegenerative disorders. The discovery of Rag GTPases has greatly expanded our understanding of the regulation of mTORC1 activity by amino acids, especially leucine and arginine. In addition to Rag GTPases, other factors that also contribute to the modulation of mTORC1 activity have been identified. In this review, we discuss the mechanisms of regulation of mTORC1 activity by particular amino acids.

## Background

### mTOR, mTOR complexes, and their fundamental roles in mammalian cells

**Fig. 1:**
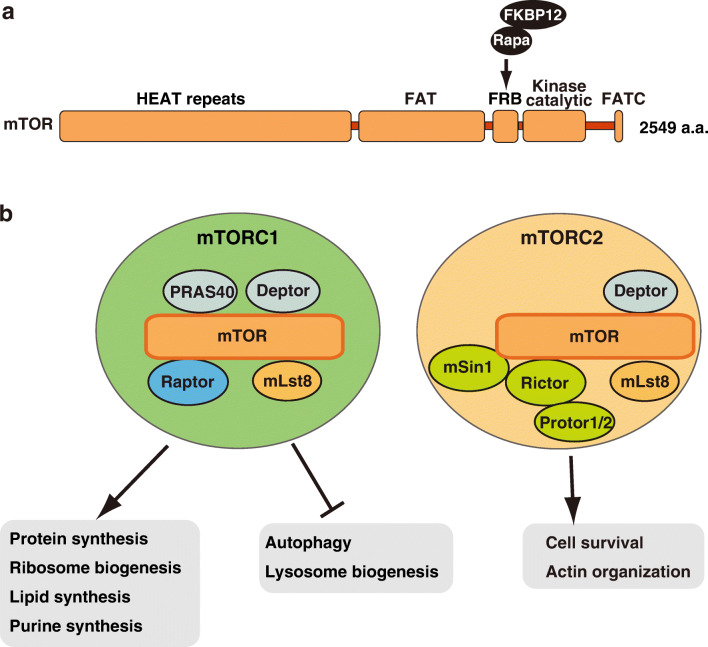
Schematic representation of the domain structure of mTOR and general functions of mTOR complexes. **a** The domain structure of mTOR. Rapamycin (Rapa), together with FKBP12, binds to the FRB domain of mTOR. The FAT and FATC domains are conserved in PIKK family members. See text for details. **b** The components of mTORC1 and mTORC2 and their general cellular functions

The target of rapamycin (TOR) was originally identified as the cellular target of the immunosuppressant and anticancer drug rapamycin in *Saccharomyces cerevisiae* [1]. TOR is an evolutionarily conserved protein in eukaryotes, from yeast to mammals. However, there are two *TOR* genes in yeast, whereas only a single *TOR* gene exists in higher eukaryotes [2]. Mechanistic TOR (mTOR), which was previously named after mammalian TOR but renamed recently, belongs to the phosphatidylinositol 3-kinase-related kinase (PIKK) family, which also includes ataxia telangiectasia mutated kinase (ATM), ataxia telangiectasia- and RAD3-related (ATR), and DNA-dependent protein kinase catalytic subunit (DNA-PKcs). mTOR possesses HEAT repeats at the N-terminal region and a FRAP-ATM-TRRAP (FAT) domain, a kinase domain, and a FAT-C-terminal (FATC) domain at the C-terminal region (Fig. 1a). Rapamycin, together with the 12-kDa FK506-binding protein (FKBP12), can bind to the FKBP12-rapamycin binding (FRB) domain located between the FAT and kinase domains and thus inhibit mTOR (Fig. 1a).

In mammalian cells, mTOR forms at least two functionally and structurally distinct complexes, the mTOR complex 1 (mTORC1) and mTORC2, in order to exert its functions (Fig. 1b). mTORC1 consists of mTOR, the regulatory associated protein of mTOR (Raptor), and mammalian LST8 homolog (mLst8) as core components. The DEP domain containing mTOR interacting protein (DEPTOR) and the proline-rich Akt substrate, 40 kDa (PRAS40) are additional components that modulate mTORC1 activity. On the other hand, mTORC2 contains mTOR, the rapamycin-insensitive companion of mTOR (Rictor), mLST8, and the mammalian stress-activated protein kinase-interacting 1 (mSin1) as core components, while DEPTOR and the protein observed with Rictor-1 and -2 (Protor-1/2) act as additional components. Raptor and Rictor are defining components of mTORC1 and mTORC2, respectively. Both mTORC1 and mTORC2 act as dimers [3–6].

mTORC1, unlike mTORC2, is sensitive to rapamycin. Indeed, recent structural analyses have shown that binding of the FKBP12-rapamycin complex to the FRB domain of mTORC1 generates steric hindrance interfering with substrate entry into the active site cleft of mTORC1 [6, 7]. In contrast, mTORC2 is resistant to acute rapamycin treatment, despite being sensitive to long-term (> 24-h) rapamycin treatment in a cell type-dependent manner [8]. Rapamycin likely inhibits mTORC2 activity by preventing nascent mTOR molecules from assembling with mTORC2-specific components such as Rictor and mSin1. Structural studies of both mTORC1 and mTORC2 by cryo-electron microscopy (cryo-EM) have revealed that Rictor and mSin1 prevent the binding of the FKBP12-rapamycin complex to the FRB domain of mTORC2 by steric hindrance, unraveling the underlying mechanism of rapamycin insensitivity of mTORC2 [6, 7, 9, 10].

Interestingly, a recent report also identified a third mTOR complex, mTORC3, resulting from the interaction between mTOR and the ETS variant transcription factor 7 (ETV7), which lacks Raptor and Rictor [11]. Furthermore, the appearance of mTORC3 seems to be triggered by ETV7, especially in cancer cells with high ETV7 expression, and might contribute to rapamycin insensitivity in many cancers.

In this review, we will mainly focus on the regulatory mechanisms of mTORC1 activity, especially those triggered by amino acids. We will first introduce the growth factor-induced mTORC1 activation pathway and then the widely accepted model of amino acid-mediated regulation of mTORC1 activity. We will also highlight potential mechanisms of amino acid-induced mTORC1 activation that are not fully understood or recognized, and yet might play significant roles as well. However, before focusing on the regulation of mTORC1 activity by growth factors and amino acids, we will briefly introduce the general functions of mTORC1 and mTORC2 in mammalian cells.

## Main text

### General functions of mTORC1

mTORC1 regulates cell growth and metabolism by modulating protein, lipid, and purine synthesis, as well as lysosome biogenesis and autophagy (Fig. 1b). mTORC1 activity is influenced by a variety of signals such as amino acids, glucose, growth factors, energy levels, and stresses [2, 12–18]. One of the key functions of mTORC1 is the promotion of anabolism. In fact, mTORC1 promotes protein synthesis by directly phosphorylating translation-related proteins, such as the ribosomal protein S6 kinase 1 (S6K1) and the eukaryotic initiation factor 4E binding protein 1 (4E-BP1). In particular, through 4E-BP proteins mTORC1 stimulates the translation of a subset of mRNAs possessing 5′ terminal oligopyrimidine (TOP) motifs or TOP-like motifs, such as mRNAs encoding ribosomal proteins [19, 20]. Thus, mTORC1 stimulates protein synthesis by both enhancing mRNA translation and upregulating ribosomal protein levels. Furthermore, mTORC1 promotes lipid synthesis by phosphorylating lipin 1, thereby increasing the activity of the sterol regulatory element-binding protein 1 (SREBP1) [21, 22], and promotes purine synthesis by stimulating the mitochondrial tetrahydrofolate (mTHF) cycle through upregulation of the methylenetetrahydrofolate dehydrogenase 2 (MTHFD2) [23]. On the other hand, mTORC1 suppresses catabolism through the inhibition of autophagy and lysosome biogenesis, which are important processes for lysosome-dependent degradation of macromolecules. Indeed, mTORC1 directly phosphorylates the unc-51 like autophagy activating kinase 1 (ULK1) [24, 25], an initial regulator of autophagy, and the transcription factor EB (TFEB), a regulator of the expression of lysosomal genes [26–29], thereby suppressing autophagy and lysosomal degradation.

### General functions of mTORC2

**Fig. 2:**
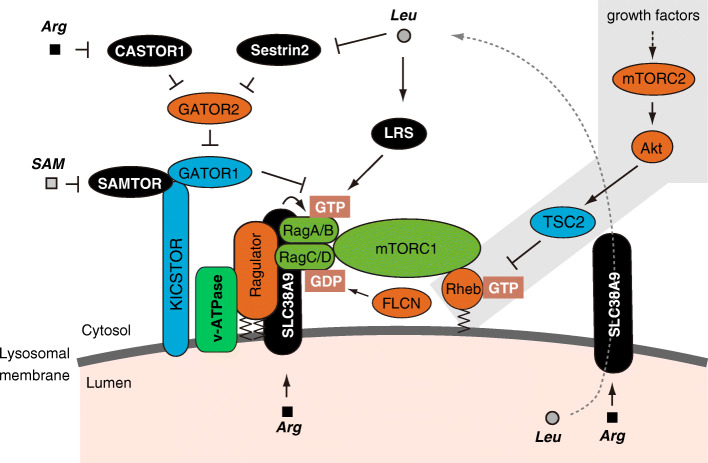
Consensus model of amino acid-dependent mTORC1 activation at the lysosome. Leucine (Leu), arginine (Arg), and S-adenosylmethionine (SAM) are sensed by cytosolic and lysosomal sensors (SESN2, LRS, CASTOR1, SLC38A9, and SAMTOR). These signals converge on Rag GTPases (heterodimeric RagA/B-RagC/D GTPases), which recruit mTORC1 onto the lysosomal surface. There mTORC1 is activated by association with the Rheb GTPase. The efflux of leucine from the lysosomal lumen to the cytosol through arginine-sensitive SLC38A9 also activates mTORC1 via cytosolic sensors

﻿Compared with mTORC1, the functions of mTORC2 are much less characterized. mTORC2 phosphorylates members of the AGC kinase family, such as Akt (also known as protein kinase B), the protein kinase C (PKC), and the serum/glucocorticoid regulated kinase 1 (SGK1), thereby regulating cell proliferation, survival, and actin cytoskeleton organization in response to growth factors [18, 30] (Fig. 1b). One of the most studied functions of mTORC2 is the phosphorylation of Akt on Ser473 [31] (Fig. 2). Previous studies have demonstrated that the association of mTORC2 with ribosomes is required for phosphorylation of Akt on Ser473 in response to insulin in vivo and in vitro [32]. In addition, the mTORC2-ribosome association was also reported to be necessary for phosphorylation of Akt on Thr450 during the translation of the Akt polypeptide, which contributes to the stabilization of Akt [33]. However, the mechanism by which the association of mTORC2 with ribosomes leads to mTORC2 activation is currently unclear. Another regulatory mechanism of mTORC2 activation has recently been reported, in which ﻿the interaction between mTOR and the pleckstrin homology (PH) domain of mSin1, an essential subunit of mTORC2, inhibits mTORC2 activity in the absence of growth factors [34]. Upon presence of growth factors, phosphatidylinositol 3-kinase (PI3K)-mediated production of phosphatidylinositol (3,4,5)-trisphosphate (PIP_3_) at the plasma membrane induces the recruitment of mTORC2 via the PH domain of mSin1, which simultaneously relieves mTORC2 inhibition and facilitates the phosphorylation of Akt on Ser473. However, Ebner et al. recently suggested that mTORC2 activity at the plasma membrane does not respond to growth factors, but rather that growth factors ﻿induce Akt phosphorylation on Ser473 by promoting the recruitment of Akt, but not of mTORC2, to the plasma membrane [35]. These authors further identified localized mTORC2 activity in mitochondria and early and late endosomes, in addition to the plasma membrane; also, they found that mTORC2 enhanced its activity in response to growth factors especially when localized in early and late endosomes, but not on the plasma membrane, although the underlying mechanism is unclear. It remains to be determined whether the mTORC2-ribosome association contributes to the modulation of mTORC2 activity in these organelles.

### Growth factor signaling-mediated activation of mTORC1

Growth factors such as insulin activate mTORC1 through class I PI3K-dependent activation of Akt (Fig. 2). Activated Akt phosphorylates and inactivates the tuberous sclerosis complex subunit 2 (TSC2, also known as tuberin). TSC2 forms the complex together with TSC1 (also known as hamartin) and the TBC1 domain family member 7 (TBC1D7) [36–39]. TSC1 and TSC2 are products of tumor suppressor genes that when mutated induce tuberous sclerosis, a genetic disorder characterized by benign tumors in multiple organs. TSC2 displays GTPase-activating protein (GAP) activity toward the small GTPase Ras homolog enriched in brain (Rheb), thereby inactivating Rheb through induction of a switch from a GTP- to a GDP-bound state. ﻿The inactivation of TSC2 by Akt leads to an increase in GTP-loaded Rheb, which is a potent direct activator of mTORC1. Thus, growth factors induce mTORC1 activation via Akt-mediated inactivation of the TSC complex, leading to Rheb activation (Fig. 2). Recent structural studies have demonstrated that GTP-bound Rheb directly binds to mTORC1 far from the kinase catalytic site and induces a conformational change to allosterically displace an auto-inhibitory element (FAT-clamp) [40, 41]. Since the lysosomal surface is the site where most mTORC1 are activated (Fig. 2, and see below), it is plausible that Rheb also resides on the lysosomal membrane to facilitate its physical interaction with mTORC1. However, the intracellular localization of Rheb is still a matter of debate [42]. In fact, several studies have demonstrated enrichment of Rheb at the lysosomes [43–47], whereas other studies have shown that Rheb is not mainly localized on lysosomal membranes but rather on ER membranes, Golgi membranes, mitochondrial outer membranes, and peroxisomes [48–55]. Interestingly, knockdown of *p53* has been reported to affect the subcellular localization of Rheb, leading to an increase in the Rheb population on lysosomal membranes [56]. In addition, unlike normal pH (pH 7.4) conditions, low pH (pH 6.3) conditions induced the dissociation of Rheb from lysosomes, where mTORC1 was still localized, with consequent mTORC1 inhibition [45]. Therefore, Rheb might not always be localized on particular membranes; rather, the localization of Rheb might fluctuate greatly depending on the cellular context. In fact, a recent report suggested that farnesylation at the C-terminal CAAX (C = cysteine, A = aliphatic, X = terminal amino acid) motif of Rheb, which mediates only weak nonselective interactions with membranes, is important for Rheb-mediated activation of mTORC1. Altogether, these results suggest that transient interactions with different membranes rather than stable interactions with a particular membrane are important for Rheb activity [50].

Initial studies proposed that the mechanism of Akt-mediated inactivation of TSC2 includes disruption of the TSC complex [57, 58] and promotion of TSC2 degradation [59]. More recent studies, however, have demonstrated that changes in TSC2 subcellular localization, rather than inhibition of the GAP activity of TSC2, play a key role in the modulation of Rheb activity [44, 60, 61]. Indeed, during starvation TSC2 is translocated from the cytoplasm to the lysosomal surface, where it inactivates lysosome-localized Rheb. Conversely, growth factors such as insulin induce the dissociation of TSC2 from lysosomes, leading to relief of Rheb suppression. However, the detailed molecular mechanism controlling TSC2 translocation remains to be determined. Previous studies have demonstrated the binding of TSC2 to 14–3-3 proteins upon TSC2 phosphorylation [58, 62–64]. Since 14–3-3 proteins often act as tethers connecting partner proteins in the cytosol, it is possible that the binding of phosphorylated TSC2 to 14–3-3 proteins is involved in the regulation of TSC2 subcellular localization.

### Consensus model of amino acid signaling for mTORC1 activation via Rag GTPases

The first step of the widely accepted model of amino acid-dependent activation of mTORC1 consists in the translocation of mTORC1 to the lysosomal surface (Fig. 2). In 2008, two independent groups pointed at Ras-related GTP binding (Rag) GTPases as the missing link between amino acid signals and mTORC1 activation [18, 43, 65]. In mammals there are four Rag proteins, forming heterodimers composed of either RagA or RagB with either RagC or RagD. Rag GTPases are tethered to lysosomal membranes through their association with the lysosome-resident pentameric Ragulator complex, which is composed of p18 (also known as LAMTOR1), p14 (LAMTOR2), MP1 (LAMTOR3), C7orf59 (LAMTOR4), and HBXIP (LAMTOR5) [66]. The Ragulator complex is associated with the lysosomal membrane via palmitoylation and myristylation of p18 [67]. The addition of amino acids triggers the shift of Rag GTPases to their active nucleotide-bound state, i.e., the GTP-bound form of RagA/B and the GDP-bound form of RagC/D. Active Rag GTPases can bind to Raptor and thereby recruit mTORC1 on the lysosomal surface, where Rheb, a direct activator of mTORC1, is also believed to reside. Finally, the association of mTORC1 with Rheb leads to mTORC1 activation (Fig. 2). This two-step activation mechanism, composed by a recruitment and an interaction step, ensures the activation of mTORC1 only when both Rag and Rheb GTPases are activated. In contrast, amino acid deprivation induces the conversion of Rag GTPases to their inactive state, i.e., GDP-bound RagA/B and GTP-bound RagC/D, which causes the release of mTORC1 from the lysosomal surface, leading to deactivation of mTORC1. Rag GTPase heterodimers display a unique structural cross talk between the two subunits [68]. Indeed, the binding of GTP to one subunit of the dimer induces a conformational change that suppresses the association of a second GTP molecule to the other subunit, possibly contributing to a rapid response to amino acid availability [68]. The key role of Rag GTPases in amino acid sensing appears to be highly conserved throughout evolution. In fact, by artificially maintaining Gtr1 (the yeast ortholog of RagA/B) in a GTP-locked state and Gtr2 (the yeast ortholog of RagC/D) in a GDP-locked state, it could at least in part mimic constitutive activation of TORC1 in *S. cerevisiae* [69, 70], although the subcellular localization of TORC1 on the vacuole (a counterpart of the lysosome in mammalian cells) was largely unchanged in response to amino acids [70].

Consistent with the crucial role of Rag GTPase, shifting between active and inactive nucleotide-bound states in controlling mTORC1 activation, some recently identified regulators of Rag GTPases, including guanine nucleotide exchange factors (GEFs) and GAPs, are involved in the regulation of the mTORC1 pathway. Importantly, Rag GTPases, and therefore mTORC1, can sense amino acid availability both in the cytosol and the lysosomal lumen via multiple mechanisms controlled by cytosol- and lysosome-localized components.

Here, we briefly describe the molecular machinery that regulates the activity of Rag GTPases (Fig. 2). It is worth noting that the consensus model of the amino acid-sensitive mechanism of mTORC1 activation has been described in many excellent reviews [18, 71, 72].

#### Key players

##### v-ATPase

The vacuolar-type H^+^-ATPase (v-ATPase) is a conserved enzyme consisting of two domains called the V_0_ and V_1_ domains and is involved in the acidification of the lysosomal lumen. Zoncu et al. demonstrated that the v-ATPase is required for mTORC1 activation in response to amino acids [73]. In fact, these authors showed that amino acid availability affected the interaction between the V_1_ domain of the v-ATPase and Ragulator-Rag GTPases, with amino acid starvation strengthening and amino acid addition weakening such interaction. Also, inhibition of v-ATPase activity by specific inhibitors or RNAi-mediated knockdown of its components prevented lysosomal translocation and activation of mTORC1, which could be rescued by the expression of the active forms of Rag GTPases. Moreover, the amino acid-sensitive interaction of Raptor with lysosomes could be reconstituted in vitro. Indeed, the addition of purified Raptor to isolated lysosomes led to amino acid-sensitive binding of Raptor to lysosomes in a v-ATPase-dependent manner. These results strongly suggest that the lysosomal lumen contains an amino acid sensing machinery working by an inside-out mechanism, although the fine details of v-ATPase-mediated regulation of mTORC1 remain unclear.

##### Ragulator

As mentioned earlier, the Ragulator complex provides a platform for lysosomal tethering of Rag GTPases [66]. The addition of amino acids hampers the interaction of Ragulator with Rag GTPases, but this phenomenon is prevented by the inhibition of v-ATPase activity [66]. Thus, the effect of Ragulator on RagA/B depends on v-ATPase activity. The initial experiment suggested that Ragulator acts as a GEF toward RagA/B to control its nucleotide-bound state [66]. However, recent biochemical studies have demonstrated that Ragulator instead promotes GTP release from RagC, relieving Rag heterodimers from locked inactive states, and then SLC38A9 (see below) acts as a GEF toward RagA to form active nucleotide-bound states of Rag heterodimers [74]. The Ragulator-Rag GTPase axis appears to be conserved in *S. cerevisiae* in the form of the Ego1-Ego2-Ego3 ternary complex (EGO-TC)-Gtr1/Gtr2 axis. Indeed, recent structural analyses have shown that the fundamental structure of the Ragulator-Rag GTPases and the EGO-TC-Gtr1/Gtr2 complexes is conserved [75–80]. EGO-TC does not exhibit any GEF activity toward Gtr1. However, it is interesting to determine whether EGO-TC has an ability to release GTP from Gtr2, given a recent finding of Ragulator actions to release GTP from RagC [74]. Vam6 (also known as Vps39) has been identified as a GEF for Gtr1 in *S. cerevisiae* [70]. Thus, it is possible that EGO-TC and Vam6 might employ a similar mechanism for heterodimeric Gtr1-Gtr2 activation in *S. cerevisiae*.

##### GATOR complex

In 2013, ﻿Bar-Peled et al. identified the GTPase activating proteins toward Rags (GATOR) complex as displaying GAP activity toward RagA/B [81]. GATOR can be divided into two multiprotein subcomplexes, called GATOR1 and GATOR2. GATOR1 is composed of Npr2-like (Nprl2), Nprl3, and DEP domain containing 5 (DEPDC5), while GATOR2 is composed of Mios, SEH1 like nucleoporin (Seh1L), SEC13 homolog (Sec13), WD repeat domain 24 (WDR24), and WDR59. GATOR1 displays GAP activity toward RagA/B and thus functions as a negative regulator of mTORC1 signaling. On the other hand, GATOR2 has been suggested to inhibit GATOR1 activity. Consistently, knockdown of GATOR2 components impaired amino acid-induced mTORC1 activation. The Seh1-associated (SEA) complex of *S. cerevisiae*, an ortholog of the GATOR complex, also acts as a GAP for Gtr1 [82–85] and consists of two subcomplexes, the Seh1-associated complex inhibiting TORC1 (SEACIT) and the Seh1-associated complex activating TORC1 (SEACAT). ﻿SEACIT is composed of the proteins Seh1-associated 1 (Sea1), Nitrogen permease regulator 2 (Npr2), and Npr3, while SEACAT contains the proteins Sea2, Sea3, Sea4, Seh1, and Sec13. However, the SEA complex consists of a single multiprotein complex, unlike the GATOR complex, in which GATOR1 and GATOR2 exist independently [84].

Recently, a protein complex called KICSTOR, composed of KPTN, ITFG2, C12orf66, and SZT2, was identified. This complex is located on the lysosome and recruits GATOR1 to the lysosomal surface [86, 87]. KICSTOR is required for the interaction of GATOR1 with RagA/B and GATOR2 and negatively regulates mTORC1 activity. Similarly, it has been demonstrated that two E3 ubiquitin ligases, RNF152 and Skp2, promote Lys63-linked polyubiquitination of RagA, leading to increased interaction with GATOR1, and thus function as negative regulators of mTORC1 activation in response to amino acids [88, 89].

##### Folliculin

The folliculin (FLCN) complex is composed of FLCN and folliculin interacting proteins 1 and 2 (FNIP1/2) and acts as a GAP for RagC/D, leading to lysosomal translocation and activation of mTORC1 [90, 91]. Upon amino acid starvation, the FLCN complex is recruited to the lysosome through binding to GDP-bound RagA/B [92]. Recent cryo-EM structural analyses have suggested that nucleotide exchange in RagA upon amino acid stimulation promotes conformational change of the lysosomal FLCN complex, thus potentiating its GAP activity toward RagC/D [93, 94] and stimulating the transition of Rag GTPases to active nucleotide-bound forms (GTP-bound RagA/B and GDP-bound RagC/D). Interestingly, the function of the FLCN complex appears to be conserved in *S. cerevisiae*. Indeed, the Lst4-Lst7 complex, an ortholog of the FLCN complex, also functions as a GAP for Gtr2 [95]. Similar to the behavior of the FLCN complex in response to amino acids, the Lst4-Lst7 complex resides at the vacuolar membrane in amino acid-deprived conditions, but transiently binds and activates Gtr2 upon amino acid stimulation [95]. Thus, amino acid sensing machineries transducing the signal through Rag GTPases, Ragulator, GATOR, and the FLCN complex most likely represent conserved and fundamental elements of mTORC1 signaling throughout evolution.

#### Other players

##### MAP4K3

The mitogen-activated protein kinase kinase kinase kinase 3 (MAP4K3), a conserved Ser/Thr kinase, was originally identified in an RNAi screen for kinases affecting mTOR signaling [96]. Knockdown of *MAP4K3* hampered amino acid-induced activation of mTORC1. In addition, the kinase activity of MAP4K3 was regulated by amino acids, but not by insulin, suggesting that MAP4K3 regulates mTORC1 activity specifically in response to amino acids. Further studies have demonstrated that MAP4K3 autophosphorylates on Ser170 in *trans*, whereas amino acid deprivation induces Ser170 dephosphorylation through protein phosphatase 2A (PP2A) containing the PR61ε-targeting subunit (PP2A_T61ε_), leading to dampening of MAP4K3 activity [97]. Consistently, knockdown of *PP2A*_*T61ε*_ resulted in higher mTORC1 activity under amino acid starvation. Importantly, MAP4K3-dependent activation of mTORC1 signaling was attenuated by *RAGC/D* knockdown, suggesting that MAP4K3 acts upstream of Rag GTPases. However, the exact mechanism by which MAP4K3 contributes to mTORC1 activation in response to amino acids remains to be fully elucidated.

##### p62

p62 (also known as sequestosome 1, or SQSTM1) is an adapter protein playing important roles in several cell functions. Duran et al. have shown that p62 interacts with Raptor and Rag GTPases in an amino acid-dependent manner [98]. In addition, p62 is located on the lysosome and facilitates lysosomal translocation of mTORC1 via its binding to Raptor and Rag GTPases. Intriguingly, p62 recruits the TNF receptor associated factor 6 (TRAF6) to mTORC1 in response to amino acids. TRAF6 is required for lysosomal translocation and activation of mTORC1 by catalyzing Lys63-linked polyubiquitination of mTOR [99]. Furthermore, p62 is phosphorylated on Thr262 and Ser272 by p38δ via an amino acid-dependent MEKK3-MEK3/6-p38δ signaling cascade [100]. The phosphorylation of p62 promotes its association with TRAF6, thus leading to activation of mTORC1 in response to amino acids.

##### GPR137B

The G protein-coupled receptor 137B (GPR137B) is a previously uncharacterized lysosome-localized G protein-coupled receptor (GPCR)-like transmembrane protein. Through genome-wide siRNA screening, Gan et al. recently identified GPR137B as a positive regulator of mTORC1 activity constitutively binding Rag GTPases [101]. Indeed, increased *GPR137B* expression induced lysosomal localization of RagA and mTORC1, whereas knockdown of *GPR137B* abrogated the mTORC1-RagA interaction and mTORC1 activation in response to amino acids. Interestingly, these authors also found that GPR137B regulated the dissociation of Rag GTPases from the lysosome, accelerating their exchange rate in response to amino acids. Similarly, Lawrence et al. have recently demonstrated that amino acids destabilize the binding of Rag GTPases to Ragulator, causing dissociation of Rag GTPases from the lysosome to curb mTORC1 activation [102]. Thus, the presence of amino acids and/or GPR137B expression levels can influence the turnover of Rag GTPases at the lysosome, possibly providing an additional layer of mTORC1 regulation. However, the ultimate fate of Rag GTPase-bound mTORC1 upon dissociation from the lysosome, i.e., whether mTORC1 is active or inactive, remains unclear.

##### EP300

Recently, leucine was reported to activate mTORC1 via the induction of Raptor acetylation on Lys1097 in some cell types [103]. The coupling of leucine levels with mTORC1 activity occurs through cytosolic acetyl coenzyme A (AcCoA), a final leucine metabolite. Indeed, the histone acetyltransferase p300 (EP300) is responsible for the acetylation of Raptor in response to AcCoA levels. The acetylation of Raptor appears to affect its interaction with Rag GTPases, thus promoting lysosomal translocation of mTORC1 [103].

#### Sensors for leucine, arginine, and S-adenosylmethionine

##### Leucyl-tRNA synthetase

The leucyl-tRNA synthetase 1 (LRS) is involved in the attachment of leucine to its cognate tRNA. Furthermore, Han et al. demonstrated that LRS displays GAP activity toward RagD, inducing the shift of RagD to its GDP-bound active form, and therefore acts as an intracellular leucine sensor for mTORC1 activation [104], although others have failed to detect GAP activity of purified LRS [90]. Leucine promotes lysosomal translocation of LRS via its interaction with RagD to activate mTORC1 [105, 106]. Although the function of the *S. cerevisiae* LRS ortholog (LeuRS) significantly differs from that of LRS, LeuRS also appears to be involved in leucine-mediated activation of TORC1. Indeed, in *S. cerevisiae*, LeuRS was shown to interact with Gtr1 (the ortholog of RagA/B) via its editing domain in a leucine-dependent manner, thus likely maintaining GTP-loaded Gtr1 to activate TORC1 [69]. Intriguingly, LRS also appears to regulate mTORC1 activity through another mechanism. In fact, LRS has been reported to induce leucylation of RagA on Lys142 or of RagB on Lys203 in response to leucine supply [107]. The leucylation of RagA/B resulted in increased GTP loading of RagA/B, leading to mTORC1 activation. Thus, aminoacyl-tRNA synthetases can also act as direct intracellular amino acid sensors to modify specific protein residues and thereby maintain cellular homeostasis through aminoacylation.

##### Sestrins

﻿Sestrins (SESNs) are highly conserved stress-inducible proteins, with three paralogs (SESN1, 2, and 3) in mammals. An earlier study suggested that p53-mediated induction of *SESN1/2* by genotoxic stress inhibits mTORC1 through the activation of the AMP-activated protein kinase (AMPK) and TSC2 [108]. On the other hand, recent studies have revealed that SESN2 acts upstream of GATOR2 as a direct cytosolic leucine sensor to regulate mTORC1 activity [109–113]. Indeed, in leucine-starved conditions, SESN2 interacts with and likely inhibits GATOR2. Instead, when leucine is present, this amino acid directly binds to SESN2, inducing the dissociation of SESN2 from GATOR2 and leading to GATOR2-mediated inhibition of GATOR1 and to subsequent mTORC1 activation. SESN2 was reported to bind to leucine with a dissociation constant (K_d_) of ~ 20 μM [109], which lies within a physiologically relevant range of cellular leucine concentration.

##### CASTOR proteins

The cytosolic arginine sensor for mTORC1 subunit 1 (CASTOR1) and CASTOR2 have been identified as binding proteins of GATOR2 components. CASTOR1 forms homodimers or heterodimers with CASTOR2 and directly binds to arginine with a K_d_ of 30 μM [114–118]. Similar to SESN2, the binding of CASTOR1 to GATOR2 likely inhibits GATOR2. In presence of arginine, direct binding of this amino acid to CASTOR1 induces the dissociation of CASTOR1 from GATOR2, leading to mTORC1 activation. Unexpectedly, recent structural analyses of CASTOR1 have suggested that arginine-bound and arginine-free CASTOR1 conformations are quite similar, except for two missing loops in the apo structure, suggesting that arginine binding to CASTOR1 might induce only small conformational changes involving these two loops [116]*.*

##### SAMTOR

The S-adenosylmethionine sensor upstream of mTORC1 (SAMTOR), a previously uncharacterized protein, was recently shown to bind to GATOR1 and KICSTOR, thus inhibiting mTORC1 activity [119]. Direct binding of S-adenosylmethionine (SAM) to SAMTOR abrogates the interaction of SAMTOR with GATOR1 and KICSTOR. Conversely, methionine starvation reduces SAM levels and induces the binding of SAMTOR to GATOR1, leading to the inhibition of mTORC1 signaling. SAMTOR was reported to bind to SAM with a K_d_ of 7 μM [119].

##### SLC38A9

The solute carrier family 38 member 9 (SLC38A9), a lysosomal membrane-resident protein with homology to amino acid transporters, was originally identified as a Rag GTPase- and Ragulator-interacting protein, and was shown to act as a positive regulator of mTORC1 signaling [120–122]. Recent studies have further demonstrated that SLC38A9 is a lysosomal arginine sensor, transporting essential amino acids including leucine from the lysosomal lumen to the cytosol in an arginine-dependent manner [123]. In addition, arginine promotes the interaction of SLC38A9 with Ragulator and Rag GTPases to activate mTORC1. As mentioned in the *Ragulator* section, SLC38A9 was reported to function as a GEF for RagA [74]. Interestingly, SLC38A9 was also shown to be required for mTORC1 activation by lysosomal cholesterol through conserved cholesterol-responsive motifs [124].

### Reevaluation of the role of individual amino acids in mTORC1 activation

Very recently, Meng et al. reevaluated the ability of individual amino acids to activate mTORC1 and found that 10 amino acids, namely alanine, arginine, asparagine, glutamine, histidine, leucine, methionine, serine, threonine, and valine, were able to promote mTORC1 activity in both murine embryonic fibroblasts and human embryonic kidney (HEK) 293A cells, although the time course of mTORC1 activation by individual amino acids differed considerably [125]. For example, leucine, arginine, and methionine, which are known to potently activate mTORC1, promoted S6K1 phosphorylation very rapidly (~ 15 min), whereas glutamine did it relatively slowly (~ 60 min). These authors could also classify these 10 amino acids into two groups, according to whether they acted through Rag GTPases-dependent or -independent pathways. Out of the 10 amino acids, glutamine and asparagine activated mTORC1 in a Rag-independent manner, but in an ADP-ribosylation factor 1 (Arf1) GTPase-dependent manner (see below).

### Glutamine-dependent activation of mTORC1 via Arf1 in the Golgi apparatus

**Fig. 3:**
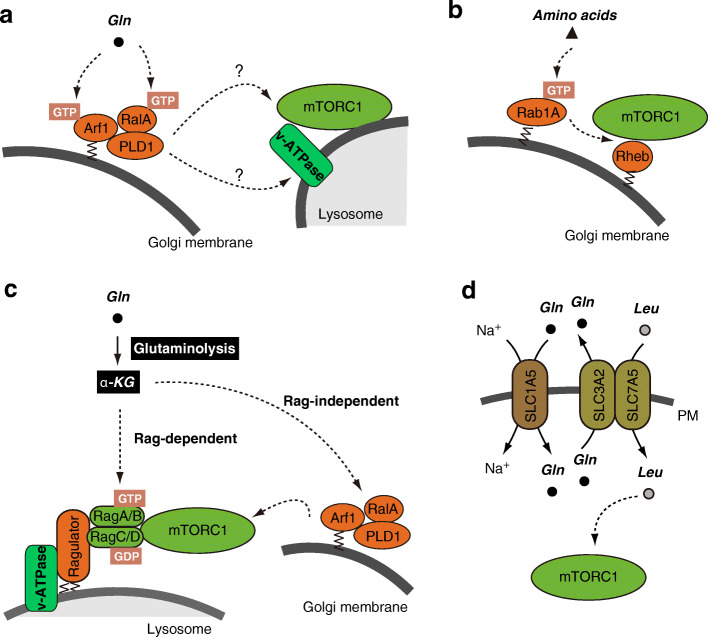
Glutamine-induced activation of mTORC1. **a** Glutamine activates mTORC1 via the Golgi-localized Arf1 GTPase. Arf1 forms a complex with the RalA GTPase and PLD1 to activate PLD1. Glutamine promotes Arf1-mediated lysosomal translocation of mTORC1 via v-ATPase, although the mechanism remains unclear. **b** Amino acids also activate mTORC1 via Golgi-localized Rab1A. **c** Glutamine is converted to α-KG via glutaminolysis. α-KG can activate mTORC1 in a Rag GTPase-dependent or a Arf1-dependent manner, but the detailed mechanisms are almost completely unknown. **d** Glutamine is required for leucine uptake through plasma membrane transporters. Glutamine enters the cytosol through SLC1A5. Cytosolic glutamine flows out of the cell in exchange for leucine via the SLC3A2-SLC7A5 anti-transporter. The glutamine-leucine antiport constitutes a rate-limiting step for mTORC1 activation

﻿Glutamine is an important amino acid consumed by cells, especially cancer cells, to meet their energy requirements, since it can provide carbon and/or nitrogen for protein, lipid, and nucleotide biosynthesis. In 2015, Jewell et al. found that glutamine activated mTORC1 in the absence of Rag GTPases [126]. Instead, glutamine requires Arf1, a Golgi-localized small GTPase, and the v-ATPase to promote lysosomal translocation and activation of mTORC1 (Fig. 3a). Although the mechanisms by which Arf1 induces mTORC1 translocation to the lysosomes are largely unknown, a recent report suggested that phosphatidic acid (PA) generated by the phospholipase D1 (PLD1) acts downstream of Arf1 to promote mTORC1 activation [127]. Indeed, PLD1 activity appears to be regulated by leucine and more potently by glutamine. Since PLD1 is activated by direct interaction with Arf1 and RalA GTPases [128, 129], we can speculate that Arf1 binding to PLD1-RalA promotes mTORC1 activation (Fig. 3a). On the other hand, GTP loading of Arf1 per se is unlikely to be involved in PLD1 activation by glutamine because it could not be triggered by glutamine and leucine availability [126]. In contrast, treatment with brefeldin A (BFA), which targets GEFs acting on Arf1, inhibited mTORC1 activation by glutamine, suggesting that nucleotide cycling of Arf1 is important for mTORC1 activation by glutamine [126].

PA produced by PLD1 appears to act as an important, closely upstream mediator of mTORC1 activation. In fact, although *RHEB* knockdown resulted in decreased PLD1 and mTORC1 activity, exogenous addition of PA could rescue decreased S6K1 phosphorylation in *RHEB* knockdown cells, suggesting that PA acts downstream of Rheb [127]. Indeed, previous reports suggested that PA directly binds to mTORC1, thus promoting its kinase activity in vitro [130], and induces the dissociation of the inhibitory subunit DEPTOR from mTORC1 to promote the activation of the complex [131]. Thus, Rheb might activate mTORC1 through two distinct mechanisms: promotion of PA production by PLD1 and direct binding to mTORC1. Interestingly, RalA was also reported to be activated by amino acids; in addition, constitutively active RalA could induce mTORC1 activation in *RHEB* knockdown cells [132], suggesting that RalA promotes mTORC1 activation downstream of Rheb. Although it remains unknown whether RalA activity is regulated by glutamine availability, it is possible that glutamine promotes PLD1 activation through GTP loading of RalA and consequent recruitment of Arf1 to form the PLD1-RalA-Arf1 ternary complex [128]. Perhaps, increases in both GTP loading of RalA and nucleotide cycling of Arf1 might be required for glutamine to activate PLD1 and mTORC1 (Fig. 3a). Even if this was the case, how PLD1-driven PA production causes lysosomal translocation of mTORC1 remains an open question. It is also worth noting that Arf1-mediated activation of mTORC1 by glutamine is relatively slow (~ 60 min) compared with leucine (~ 15 min). Thus, it is possible that, in presence of a variety of amino acids, leucine and arginine trigger a rapid response modulating mTORC1 activity in a Rag GTPase-dependent manner, while glutamine regulates mTORC1 activity in an Arf1-dependent manner.

In addition to the involvement of the Golgi-localized Arf1, a role of the Golgi-localized Rab1A GTPase in mTORC1 activation has also been suggested [55]. Amino acids promote GTP loading of Rab1A, which stimulates Rheb-mTORC1 association at the Golgi (Fig. 3b). It remains to be determined whether Rab1A perceives glutamine and whether the two GTPases Rab1A and Arf1 communicate with each other to regulate mTORC1 activity in the Golgi.

In yeast, glutamine also activates TORC1 independently from the Gtr1/Gtr2-EGO axis, the counterpart of the Rag GTPase-Ragulator axis. Indeed, several recent reports have demonstrated that Pib2, a FYVE domain-containing protein, acts to relay glutamine availability to TORC1 at the vacuoles [133–135], although the mechanism of Pib2-promoted TORC1 activation remains unclear. Consistent with the presence of the FYVE domain within Pib2, which binds to phosphatidylinositol 3-phosphate (PI3P), loss of PI3P production in *Δvps34* cells hampered the activation of TORC1 in response to glutamine [133, 134], suggesting that PI3P-mediated vacuolar localization of Pib2 supports TORC1 activation by glutamine. In addition, the interaction between Pib2 and TORC1 became stronger in response to glutamine. However, a recent study demonstrated that knockdown of either the mammalian ortholog of *PIB2*, *LARP (also known as Phafin1)* or *R3H domain and coiled-coil containing 1* (*R3HCC1*), which display a region similar to the TORC1-interacting domain of Pib2 (motif E), did not affect glutamine-induced activation of mTORC1 [125], suggesting that the function of Pib2 in relaying glutamine availability to TORC1 is unlikely to be conserved in mammals.

Glutamine was also reported to activate mTORC1 via its metabolic conversion to α-ketoglutarate (α-KG) by glutaminolysis (Fig. 3c) [136], occurring through two deamination steps. First, a glutaminase metabolizes glutamine to glutamate; subsequently, a glutamate dehydrogenase, two different transaminases, a glutamate oxaloacetate transaminase, and a glutamate pyruvate deaminase metabolize glutamate to α-KG. The resulting increase in α-KG levels promotes lysosomal translocation and activation of mTORC1 by stimulating GTP loading of RagB [136]. On the other hand, a recent study suggested that the addition of dimethyl-α-KG (DM-α-KG), a cell-permeable analog of α-KG, promotes mTORC1 activation even in *RAGA/B* knockout cells. Moreover, the effect of DM-α-KG on mTORC1 was counteracted by PLD1 inhibition, indicating that α-KG stimulates mTORC1 via the PLD1-RalA-Arf1 axis in addition to RagB [127].

Finally, glutamine indirectly participates in mTORC1 activation by acting as the key amino acid for the uptake of leucine (Fig. 3d). The solute carrier family 1 member 5 (SLC1A5), a high-affinity transporter for neutral amino acids, is required for glutamine uptake. On the other hand, glutamine is exported out of cells in exchange for leucine through SLC7A5/SLC3A2, a heterodimeric antiporter, thus creating a rate-limiting step for mTORC1 activation [137].

### Relationship between intracellular Ca^2+^ mobilization and mTORC1 signaling

**Fig. 4:**
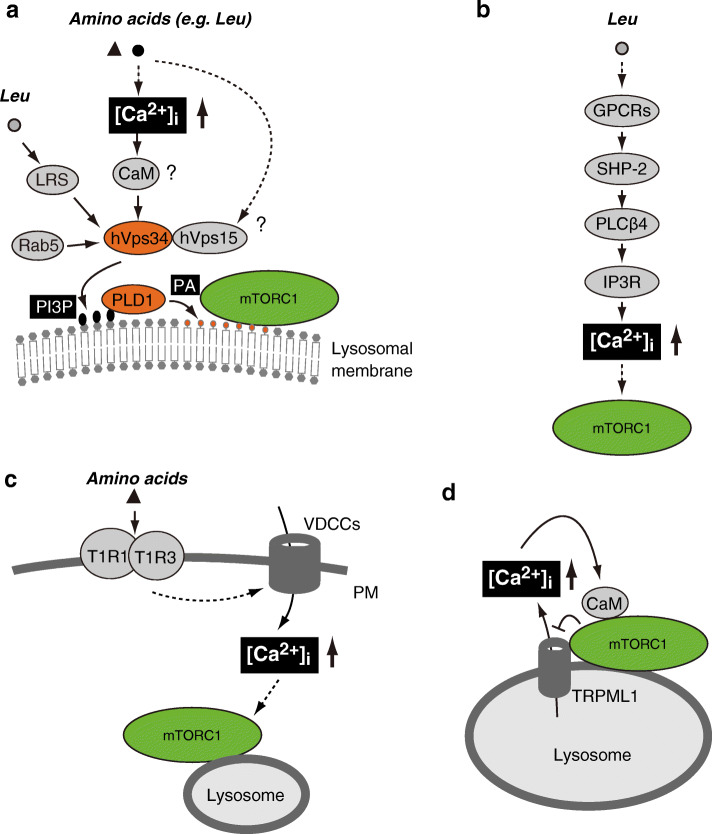
Ca^2+^-mediated pathways of mTORC1 activation. **a** Leucine triggers the increase of intracellular Ca^2+^ concentrations ([Ca^2+^]_i_) and stimulates the kinase activity of hVps34. The rise in [Ca^2+^]_i_ may be sensed by CaM. Leucine may also activate hVps34 via hVps15 and LRS. PI3P production by activated hVps34 results in recruitment of PLD1, producing PA, **b** Leucine may be sensed by GPCRs, which elevate [Ca^2+^]_i_ through Ca^2+^ fluxes from the ER via SHP-2-PLCβ4-IP3R signaling, leading to mTORC1 activation. **c** The umami receptor, T1R1/T1R3, senses amino acids and increases [Ca^2+^]_i_ in a VDCC-dependent manner via unknown mechanisms, leading to lysosomal translocation and activation of mTORC1. **d** TRPML1 mediates lysosomal Ca^2+^ release into the cytosol, resulting in a local and transient increase in [Ca^2+^]_i_, which might be sensed by CaM to activate mTORC1. In turn mTORC1 phosphorylates TRPML1 to negatively regulate its Ca^2+^ release activity. Such feedback loop possibly maintains lysosomal and cellular homeostasis during nutrient starvation

Despite some unknown aspects of this regulatory mechanism, it is well-established that mTORC1 activity can be regulated by intracellular Ca^2+^ mobilization. In fact, more than two decades ago, Graves et al. reported that treatment with Ca^2+^ ionophore A23187 or thapsigargin, increasing intracellular Ca^2+^ concentration, activated S6K1, while this effect was avoided by pretreatment with a Ca^2+^ chelator, such as EGTA or BAPTA-AM [138]. Similarly, Conus et al. reported that intracellular Ca^2+^ levels affected the phosphorylation status of S6K1 but not Akt in response to mitogenic stimulation [139]. Indeed, a decrease in intracellular Ca^2+^ concentration by pretreatment with the Ca^2+^ chelators EGTA or BAPTA prevented the activation of S6K1 but not Akt. On the other hand, an increase in intracellular Ca^2+^ concentration induced by ionomycin or thapsigargin treatment triggered the hyperphosphorylation of S6K1, but little or no activation of Akt. Thus, intracellular Ca^2+^ concentrations preferentially affect S6K1 activation. Interestingly, it was reported that the addition of amino acids, especially leucine, to amino acid-starved HeLa cells resulted in an increased intracellular Ca^2+^ concentration [140] (Fig. 4a). Mechanistically, it was demonstrated that amino acid-induced high intracellular Ca^2+^ levels lead to the direct binding of Ca^2+^/calmodulin (CaM) to the human Vps34 (hVps34), which triggers the production of PI3P from phosphatidyl inositol (Fig. 4a). Consistently, amino acids or glucose availability were also reported to influence the activity of hVps34, with subsequent S6K1 activation [141, 142]. However, even though initial experiments identified hVps34 as a potential mediator of Ca^2+^ signaling in response to amino acids, the involvement of Ca^2+^/CaM in the regulation of hVps34 activity remains a matter of debate [143]. In fact, Yan et al. demonstrated that hVps34 activity is regulated by its interaction with hVps15, a subunit of hVps34 kinase, but not by Ca^2+^/CaM, despite the fact that CaM could bind hVps34 [143]. Thus, a consistent rationale for nutrient-dependent regulation of hVps34 activity by CaM has not yet been provided. More recently, LRS has been shown to mediate leucine-dependent activation of hVps34 [144]. In fact, LRS (aa 361–720) interacted with hVps34 and promoted its kinase activity in vitro in a leucine-dependent manner. Several other reports have also confirmed that the production of PI3P by hVps34 is indeed involved in mTORC1 activation, especially by amino acids. For example, increased PI3P levels through hVps34 activation triggered the recruitment of PLD1 to lysosomes via its Phox Homology domain, which in turn induced PA production and subsequent mTORC1 activation (Fig. 4a) [144, 145]. Finally, the Rab5A GTPase, a regulator of PI3P production by hVps34 [146], is likely involved in mTORC1 activation because Rab5A activity is required for mTORC1 activation in response to both amino acids and growth factors [147, 148].

In C2C12 myoblasts, leucine administration was reported to induce increased intracellular Ca^2+^ concentrations through release from the ER Ca^2+^ store in an inositol-1,4,5-trisphosphate receptor (IP3R)-dependent manner (Fig. 4b). The release of Ca^2+^ via IP3R appears to be mediated by ﻿the SH2 domain-containing protein tyrosine phosphatase (SHP-2)-phospholipase C beta 4 (PLCβ4) axis, although it remains unknown how leucine signals are transduced to SHP-2 [149]. Nevertheless, the tyrosine phosphatase activity of SHP-2 is essential for its activity, linking leucine availability with ER Ca^2+^ release and S6K1 phosphorylation. In addition, leucine-induced cytosolic Ca^2+^ accumulation is sensitive to the pertussis toxin, which inhibits the G_i_α subunit of heterotrimeric G-proteins. These results suggest that leucine is sensed by a type of GPCR. In this regard, it is interesting to note that the umami taste 1 receptor T1R1/T1R3, a heterodimeric GPCR complex, can sense amino acid availability to regulate mTORC1 in a variety of cell lines (Fig. 4c) [150]. Indeed, PLCβ activity appears to be involved in T1R1/T1R3-mediated mTORC1 activation [150, 151]. Moreover, amino acid-mediated activation of T1R1/T1R3 increases intracellular Ca^2+^ concentration, at least in part, through L-type voltage-dependent Ca^2+^ channels (VDCCs), thus activating mTORC1. Indeed, pretreatment with nifedipine, a VDCC inhibitor, reduced mTORC1 activation by amino acids [150]. However, it remains unclear how T1R1/T1R3 activates VDCCs and regulates mTORC1. Although *T1R3* knockdown resulted in reduced lysosomal translocation of mTOR in response to amino acids, it remains to be determined whether inhibition of Ca^2+^ entry through VDCCs led to a decrease in lysosomal localization of mTOR.

### Lysosomal Ca^2+^ release and mTORC1

In addition to the ER, lysosomes also act as intracellular Ca^2+^ stores. Lysosomal Ca^2+^ can influence mTORC1 activity, especially under stressful conditions [152, 153]. A lysosomal Ca^2+^ channel, TRPML1 (also known as mucolipin 1), might represent the missing link between lysosomal Ca^2+^ release and mTORC1 regulation in a context-dependent manner (Fig. 4d) [152–155], although it has also been suggested that TRPML1 does not affect mTORC1 activity [156, 157]. Nevertheless, pharmacological or RNAi-mediated inhibition of TRPML1 resulted in reduced mTORC1 activity, whereas activation of TRPML1 by treatment with an agonist or *TRPML1* overexpression retained to some extent mTORC1 activity during starvation [152]. Interestingly, knockdown of *TRPML1* reduced the lysosomal translocation of mTOR. In addition, lysosomal Ca^2+^ release via TRPML1 after treatment with the ML-SA1 agonist also induced lysosomal translocation of mTOR even in starved condition, while mTOR translocation was almost completely abolished by treatment with the Ca^2+^ chelator BAPTA-AM. These results imply that TRPML1-mediated lysosomal Ca^2+^ release leads to mTORC1 activation by regulating the subcellular localization of mTOR. However, BAPTA-AM treatment or knockdown of *TRPML1* still resulted in decreased mTORC1 activity even in the presence of constitutively active RagB (RagB^Q99L^), which forced mTORC1 translocation to the lysosome. This phenomenon indicates that TRPML1-dependent Ca^2+^ release can also affect mTORC1 activity independently from the localization of mTOR. Indeed, it has been reported that TRPML1 is weakly associated with mTOR. Furthermore, CaM is also associated with mTORC1 in a Ca^2+^-dependent manner and binding of Ca^2+^/CaM to mTORC1 may increase mTORC1 kinase activity in vitro [153]. Thus, TRPML1-mediated lysosomal Ca^2+^ release appears to affect mTORC1 activity through several different mechanisms. Moreover, it has been reported that transient nutrient starvation can induce TRPML1-mediated lysosomal Ca^2+^ release [152, 156]. Because mTORC1 has been reported to phosphorylate TRPML1 on Ser572 and Ser576, thus decreasing its Ca^2+^ release activity [158], it is possible that inactivation of mTORC1 under nutrient starvation can relieve TRPML1 inhibition, leading to transient Ca^2+^ release. Furthermore, TRPML1 is required for mTORC1 reactivation under prolonged starvation [152]. In conclusion, the reciprocal regulation between mTORC1 and TRPML1 might be important for adaptation to short-term and prolonged nutrient stresses (Fig. 4d).

### Potential transduction of amino acid signals along the TSC complex-Rheb axis

In the current model of mTORC1 activation, signals from amino acids and growth factors are independently transduced to mTORC1, as illustrated in Fig. 2. This assumption is based on studies showing that TSC2-deficient cells were still sensitive to amino acid availability and that amino acids, but not insulin, were unlikely to affect the phosphorylation status of TSC2 [141, 142, 159]. However, several reports demonstrated that amino acid availability can influence GTP loading of Rheb, implying that the TSC2-Rheb axis can be regulated in response to amino acids [47, 159–161], although contradicting reports can also be found [142, 162].

Intriguingly, recent studies have demonstrated that both amino acids and growth factors govern the intracellular localization of TSC2, thus controlling a key mechanism for the regulation of mTORC1 activity [44, 60, 61]. Specifically, amino acid deprivation induces Rag GTPase-mediated lysosomal translocation of TSC2 and thus inactivates Rheb at the lysosome [60, 61]. Moreover, arginine deprivation has been shown to induce lysosomal translocation of TSC2 [161], while addition of arginine promoted the dissociation of TSC2 from the lysosome. Arginine also disrupted the interaction of TSC2 with Rheb in vivo and in vitro. Thus, arginine likely participates in the regulation of the TSC2-Rheb axis [161]. Collectively, it appears that amino acid availability can control the activity of the TSC complex-Rheb axis as well as the GATOR-Rag GTPase axis to integrate cellular status with growth and metabolism, at least in some contexts. Future studies will be necessary to unveil the complex regulation of mTORC1 signaling required for cells to appropriately modulate their metabolism and growth.

## Conclusions

Recent biochemical and structural analyses have dramatically improved current knowledge of the molecular mechanisms of amino acid sensing, especially leucine and arginine, which ultimately converge on Rag GTPases to regulate mTORC1 activity. As shown above, it is well established that the involvement of Rag GTPases is essential for amino acid-mediated mTORC1 regulation, but much more complex regulatory mechanisms of mTORC1 have also emerged. Undoubtedly, further studies will provide new insights into how mTORC1 activity can be regulated by the integration of a variety of input signals to maintain cellular homeostasis; this knowledge will also provide novel approaches to treat human diseases, especially those associated with aberrant mTORC1 activity.

## Data Availability

Not applicable.

## Abbreviations

TOR: Target of Rapamycin
mTOR: mechanistic Target of Rapamycin
PIKK: phosphatidylinositol 3-kinase-related kinase
ATM: ataxia telangiectasia mutated kinase
ATR: ataxia telangiectasia- and RAD3-related
DNA-PKcs: DNA-dependent protein kinase catalytic subunit
FAT: FRAP-ATM-TRRAP
FATC: FAT-C-terminal
FKBP12: 12-kDa FK506-binding protein
FRB: FKBP12-rapamycin-binding
mTORC: mammalian/mechanistic Target of Rapamycin Complex
Raptor: the regulatory-associated protein of mTOR (Raptor)
mLst8: mammalian LST8 homolog
DEPTOR: DEP domain-containing mTOR-interacting protein
PRAS40: proline-rich Akt substrate, 40 kDa
Rictor: the rapamycin-insensitive companion of mTOR
mSin1: the mammalian stress-activated protein kinase-interacting 1
Protor: protein observed with Rictor
cyro-EM: cryo-electron microscopy
ETV7: ETS variant transcription factor 7
S6K1: S6 kinase 1
4E-BP1: the eukaryotic initiation factor 4E binding protein 1
TOP: terminal oligopyrimidine
SREBP1: the sterol regulatory element-binding protein 1
mTHF: mitochondrial tetrahydrofolate
MTHFD2: methylenetetrahydrofolate dehydrogenase 2
ULK1: unc-51 like autophagy activating kinase 1
TFEB: transcription factor EB
PKC: protein kinase C
SGK1: serum/glucocorticoid regulated kinases 1
PH: pleckstrin homology
PI3K: phosphatidylinositol 3-kinase
PIP_3_: phosphatidylinositol (3,4,5)-trisphosphate
TSC: tuberous sclerosis complex
TBC1D7: TBC1 domain family member 7
GAP: GTPase-activating protein
Rheb: Ras homolog enriched in brain
Rag: Ras related GTP binding
GEF: guanine nucleotide exchange factor
v-ATPase: the vacuolar-type H^+^-ATPase
EGO-TC: Ego1-Ego2-Ego3 ternary complex
GATOR: GAP activity towards the Rags
Npr2: Nitrogen permease regulator 2
Nprl2: Npr2-like
DEPDC5: DEP domain containing 5
Seh1L: SEH1 like nucleoporin
WDR: WD repeat domain
SEACIT: the SEA subcomplex inhibiting TORC1
SEACAT: the SEA subcomplex activating TORC1
FLCN: folliculin
FNIP1/2: FLCN-interacting proteins 1 and 2
MAP4K3: The mitogen-activated protein kinase kinase kinase kinase 3
PP2A: protein phosphatase 2A
PP2A_T61ε_: PR61ε-targeting subunit
TRAF6: TNF receptor associated factor 6
GPR137B: The G protein-coupled receptor 137B
GPCR: G-protein coupled receptor
EP300: the histone acetyltransferase p300
LRS: Leucyl-tRNA synthetase 1
LeuRS: LRS ortholog
SESM: Sestrin
AMPK: AMP-activated kinase
CASTORs: Cellular Arginine Sensor for mTORC1
SAMTOR: S-adenosylmethionine sensor upstream of mTORC1
SAM: S-adenosylmethionine
SLC38A9: The solute carrier family 38 member 9
Arf1: ADP-ribosylation factor 1
PA: phosphatidic acid
PLD: phospholipase D
BFA: brefeldin A
PI3P: phosphatidylinositol 3-phosphate
R3HCC1: R3H and coiled- coil domain–containing protein 1
α-KG: α-ketoglutarate
DM-α-KG: dimethyl-α-KG
CaM: calmodulin
IP3R: inositol-1, 4, 5-trisphosphate receptor
SHP-2: SH2 domain-containing protein tyrosine phosphatase
PLCβ4: phospholipase Cβ4
VDCC: L-type voltage-dependent Ca^2+^ channel
[Ca^2+^]_i_: intracellular Ca^2+^ concentration
